# Predicting Diagnostic Gene Biomarkers Associated With Immune Infiltration in Patients With Acute Myocardial Infarction

**DOI:** 10.3389/fcvm.2020.586871

**Published:** 2020-10-23

**Authors:** Enfa Zhao, Hang Xie, Yushun Zhang

**Affiliations:** Department of Structural Heart Disease, The First Affiliated Hospital of Xi'an Jiaotong University, Xi'an, China

**Keywords:** acute myocardial infarction, immune infiltration, diagnostic, biomarker, CIBERSORT

## Abstract

**Objective:** The present study was designed to identify potential diagnostic markers for acute myocardial infarction (AMI) and determine the significance of immune cell infiltration in this pathology.

**Methods:** Two publicly available gene expression profiles (GSE66360 and GSE48060 datasets) from human AMI and control samples were downloaded from the GEO database. Differentially expressed genes (DEGs) were screened between 80 AMI and 71 control samples. The LASSO regression model and support vector machine recursive feature elimination (SVM-RFE) analysis were performed to identify candidate biomarkers. The area under the receiver operating characteristic curve (AUC) value was obtained and used to evaluate discriminatory ability. The expression level and diagnostic value of the biomarkers in AMI were further validated in the GSE60993 dataset (17 AMI patients and 7 controls). The compositional patterns of the 22 types of immune cell fraction in AMI were estimated based on the merged cohorts using CIBERSORT.

**Results:** A total of 27 genes were identified. The identified DEGs were mainly involved in carbohydrate binding, Kawasaki disease, atherosclerosis, and arteriosclerotic cardiovascular disease. Gene sets related to atherosclerosis signaling, primary immunodeficiency, IL-17, and TNF signaling pathways were differentially activated in AMI compared with the control. IL1R2, IRAK3, and THBD were identified as diagnostic markers of AMI (AUC = 0.877) and validated in the GSE60993 dataset (AUC = 0.941). Immune cell infiltration analysis revealed that IL1R2, IRAK3, and THBD were correlated with M2 macrophages, neutrophils, monocytes, CD4^+^ resting memory T cells, activated natural killer (NK) cells, and gamma delta T cells.

**Conclusion:** IL1R2, IRAK3, and THBD can be used as diagnostic markers of AMI, and can provide new insights for future studies on the occurrence and the molecular mechanisms of AMI.

## Introduction

Acute myocardial infarction (AMI) is a common event in coronary heart disease that results from interrupted blood flow to a certain area of the heart. It is considered one of the primary causes of disability and death from cardiovascular disease worldwide, and is a leading health threat in humans (1). AMI remains the primary cause of morbidity and mortality worldwide, with ~7 million patients diagnosed with AMI each year (2, 3). AMI continues to be the primary cause of death in 2020. Approximately half of patients who suffer from cardiovascular diseases die from AMI (4). The rapid and accurate diagnosis of AMI is the first step to improve the clinical management and survival rate of AMI patients. A spectrum of biochemical markers have been related to the incidence of AMI and are widely used for the clinical diagnosis of AMI including the MB isoenzyme of creatine kinase (CK-MB), lactate dehydrogenase (LDH), cardiac myoglobin, and cardiac troponin I (cTnI) and T (cTnT) (5, 6). However, they are insufficient for the early detection of AMI because of limitations in sensitivity and specificity (7). Furthermore, the well-known risk factors for AMI, such as a history of smoking, obesity, high serum cholesterol, bad eating habits, diabetes, and hypertension, can only predict AMI prevention and outcomes and fall to adequately provide an acute diagnosis (8). These results demonstrate that genetic factors also play a vital role in the pathogenesis of AMI. In fact, AMI is a complex and multifactorial disease that occurs as a result of the interaction between genetic and environmental factors (9).

In recent years, microarray technology, together with integrated bioinformatics analysis, has been performed to identify novel genes related to various diseases that might act as diagnostic and prognostic biological markers (10–14). For example, the expression of FFAR2, also known as GPR43, in AMI patients has been found to be notably lower than in the controls, and low levels of FFAR2 expression in peripheral blood was confirmed as an independent risk predictor for AMI, with an odds ratio of 6.308 (15). The upregulation of the suppressor of cytokine signaling 3 (SOCS3) gene increases the risk of AMI by potentiating inflammatory responses (16). Moreover, research has shown that immune cell infiltration plays an increasingly significant role in the occurrence and development of various diseases (11, 17–19). With regards to AMI, mast cells, M2 macrophages, and eosinophils have been demonstrated to affect cardiac function after AMI, providing novel insights into the significance of immune modulation in the infarcted heart (20). However, to date, few studies have applied CIBERSORT to explore immune cell infiltration in AMI and investigate candidate diagnostic markers for AMI.

In this study, we downloaded two microarray datasets of AMI from the GEO database. The two datasets were merged into a meta-data cohort. Differentially expressed gene (DEG) analysis was performed between the AMI and controls. Machine-learning algorithms were used to filter and identify diagnostic biomarkers of AMI. Candidate genes strongly related to immune infiltration were identified and validated in another validation cohort and were used to construct the diagnostic prediction model using a logistic regression method. In this study, CIBERSORT was used for the first time to quantify the proportions of immune cells in samples of AMI and normal tissues based on their gene expression profiling. Furthermore, we explored the relationship between the identified biomarkers and infiltrating immune cells to provide a basis for further research.

## Materials and Methods

### Microarray Data

The series of matrix files of the GSE48060 and GSE66360 datasets were obtained from http://www.ncbi.nlm.nih.gov/geo/, which were both based on the GPL570 platform of Affymetrix Human Genome U133 Plus 2.0 Array. The GSE48060 dataset included 49 AMI and 50 controls collected from circulating endothelial cells, whereas the GSE66360 dataset included 31 AMI and 21 controls collected from the peripheral blood. The probes in each dataset were changed into gene symbols based on their probe annotation files. For more than one probe corresponding to the same gene symbol, the probe average was calculated as the final expression value of the gene. These two datasets were merged into a metadata cohort for further integration analysis because they have the same platform and are significant for combining data from different datasets. Furthermore, the combat function of the “SVA” package of R software was applied to remove the batch effect (21). In addition, the GSE60993 dataset, collected from peripheral blood and containing 17 AMI and 7 control samples, was used as the validation cohort using the Illumina HumanWG-6 v3.0 expression beadchip.

### Data Processing and DEG Screening

The two datasets were merged into a metadata cohort and the combat function of the SVA package was used to preprocess and remove batch effects. The limma package of R (http://www.bioconductor.org/) was used for background correction, normalization between arrays, and differential expression analysis between 80 AMI and 71 control samples. Samples with an adjusted false discovery rate *P* < 0.05 and |log fold change (FC)| > 1.2 were considered as the threshold points for DEGs.

### Functional Enrichment Analysis

Disease ontology (DO) enrichment analyses were performed on DEGs using the “clusterProfiler” and DOSE packages in R (22, 23). Gene set enrichment analysis (GSEA) was used to identify the most significant functional terms between the AMI and control groups. The “c2.cp.kegg.v7.0.symbols.gmt” from the Molecular Signatures Database (MSigDB) was used as the reference gene set. A gene set was regarded as significantly enriched if a *P* < 0.05 and false discovery rate <0.025.

### Candidate Diagnostic Biomarker Screening

To identify significant prognostic variables, two machine-learning algorithms were used to predict disease status. The least absolute shrinkage and selection operator (LASSO) is a regression analysis algorithm that uses regularization to improve the prediction accuracy. The LASSO regression algorithm was carried out using the “glmnet” package in R to identify the genes significantly associated with the discrimination of AMI and normal samples. Support vector machine (SVM) is a supervised machine-learning technique widely utilized for both classification and regression. To avoid overfitting, an RFE algorithm was employed to select the optimal genes from the meta-data cohort (24). Therefore, to identify the set of genes with the highest discriminative power, support vector machine recursive feature elimination (SVM-RFE) was applied to select the appropriate features. The overlapping genes between the two algorithms were included and the expression levels of candidate genes were further validated in the GSE60993 dataset.

### Diagnostic Value of Feature Biomarkers in AMI

To test the predictive value of the identified biomarkers, we generated an ROC curve using the mRNA expression data from 80 AMI and 71 control samples. The area under the ROC curve (AUC) value was utilized to determine the diagnostic effectiveness in discriminating AMI from control samples and further validated in the GSE60993 dataset.

### Discovery of Immune Cell Subtypes

To quantify the relative proportions of infiltrating immune cells from the gene expression profiles in AMI, a bioinformatics algorithm called CIBERSORT (https://cibersortx.stanford.edu/) was used to calculate immune cell infiltrations. The putative abundance of immune cells was estimated using a reference set with 22 types of immune cell subtypes (LM22) with 1,000 permutations (25). Correlation analysis and visualization of 22 types of infiltrating immune cells were performed using the R package “corrplot.” Violin plots were drawn using the “vioplot” package in R to visualize the differences in immune cell infiltration between the AMI and control samples.

### Correlation Analysis Between Identified Genes and Infiltrating Immune Cells

The association of the identified gene biomarkers with the levels of infiltrating immune cells was explored using Spearman's rank correlation analysis in R software. The resulting associations were visualized using the chart technique with “ggplot2” package.

### Statistical Analysis

All statistical analyses were conducted using R (version 3.6.3). Group comparisons were undertaken for continuous variables using Student's *t*-test for normally distributed variables or the Mann–Whitney *U*-test for variables with an abnormal distribution. LASSO regression analysis was carried out using the “glmnet” package, and the SVM algorithm was performed using the e1071 package in R. ROC curve analysis was used to determine the diagnostic efficacy of the diagnostic biomarkers included. The relationship between the expression of gene biomarkers and infiltrating immune cells was analyzed using Spearman's correlation. All statistical analyses were two-sided with *P* < 0.05 were regarded statistically significant.

## Results

### Identification of DEGs in AMI

**Figure 1:**
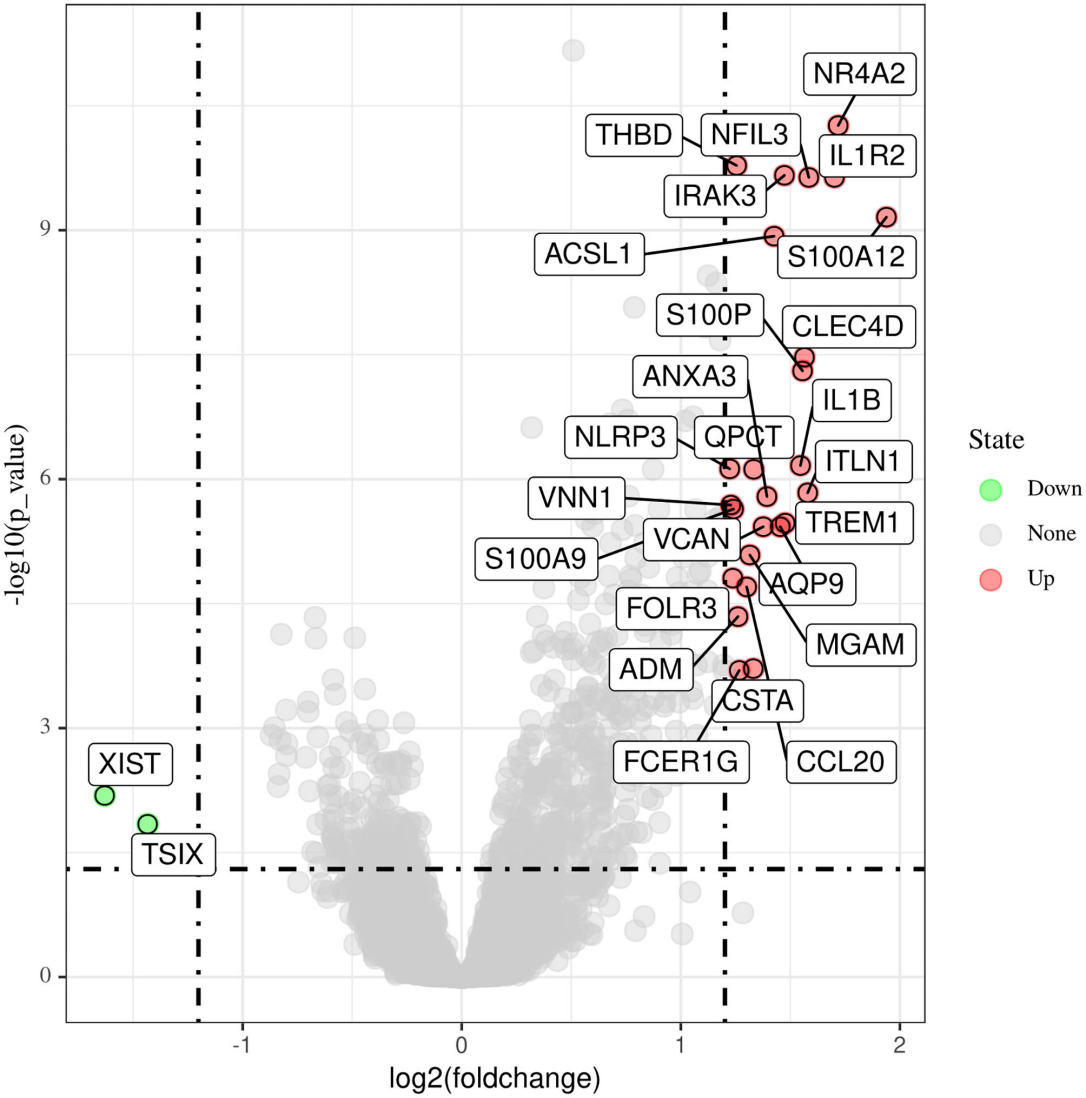
Differentially expressed genes between acute myocardial infarction tissue and control samples.

Data from a total of 80 AMI and 71 control samples from two GEO datasets (GSE66360 and GSE48060) were retrospectively analyzed in this study. The DEGs of the metadata were analyzed using the limma package after removing the batch effects. A total of 27 DEGs were obtained: 25 genes were significantly upregulated and 2 genes were significantly downregulated (Figure 1).

### Functional Correlation Analysis

**Figure 2:**
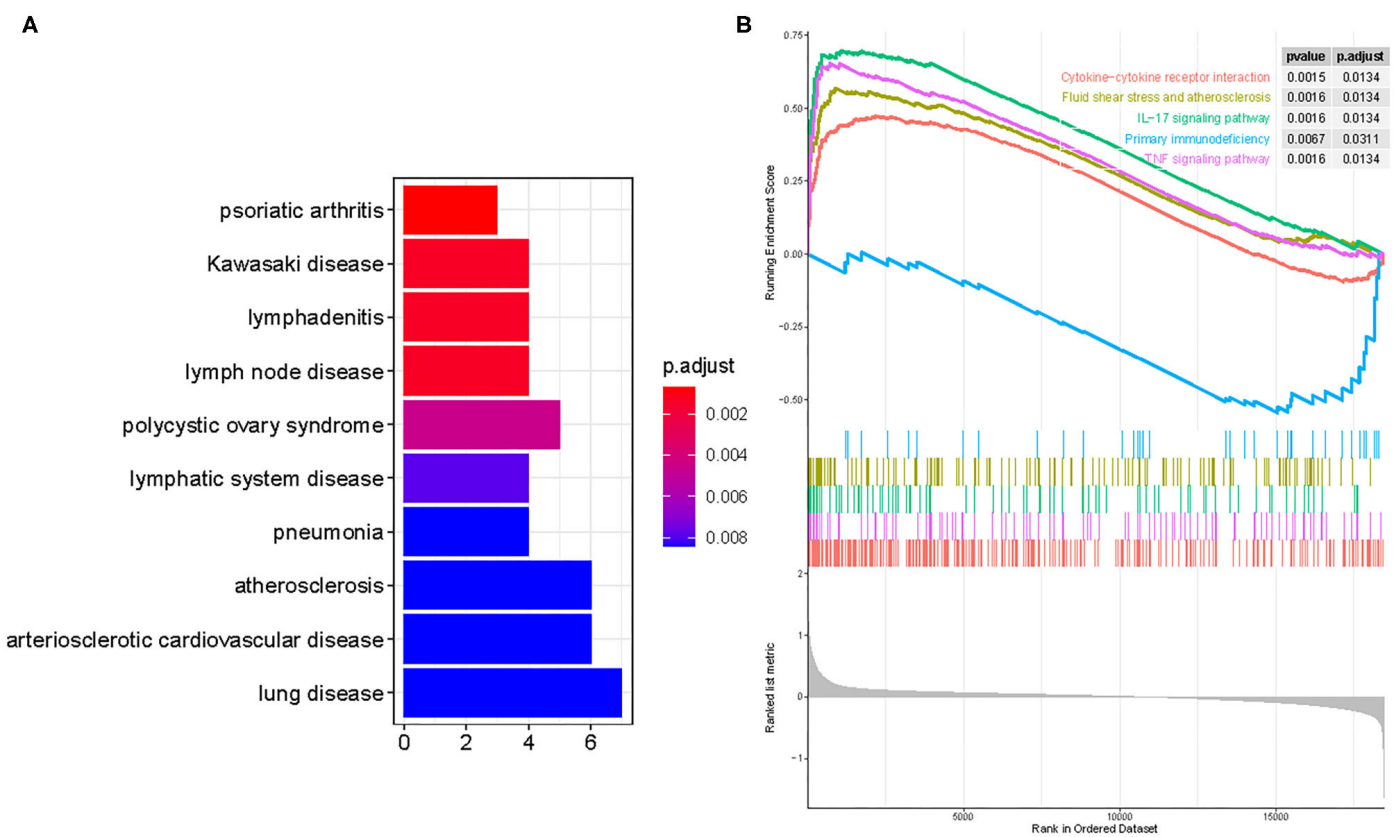
Functional enrichment analyses to identify potential biological processes via disease ontology and gene set enrichment analysis. **(A)** Disease ontology enrichment analysis of differentially expressed genes between AMI and control samples. **(B)** Enrichment analyses via gene set enrichment analysis.

DO pathway enrichment analyses were conducted to investigate the function of DEGs. The results indicated that diseases enriched by DEGs were mainly associated with arteriosclerotic cardiovascular disease, atherosclerosis, lymphadenitis, and Kawasaki disease (Figure 2A). The GSEA results demonstrated that the enriched pathways mainly involved cytokine–cytokine receptor interaction, atherosclerosis, IL-17 signaling pathway, primary immunodeficiency, and TNF signaling pathways (Figure 2B). These findings strongly suggest that the immune response plays an essential role in AMI.

### Identification and Validation of Diagnostic Feature Biomarkers

**Figure 3:**
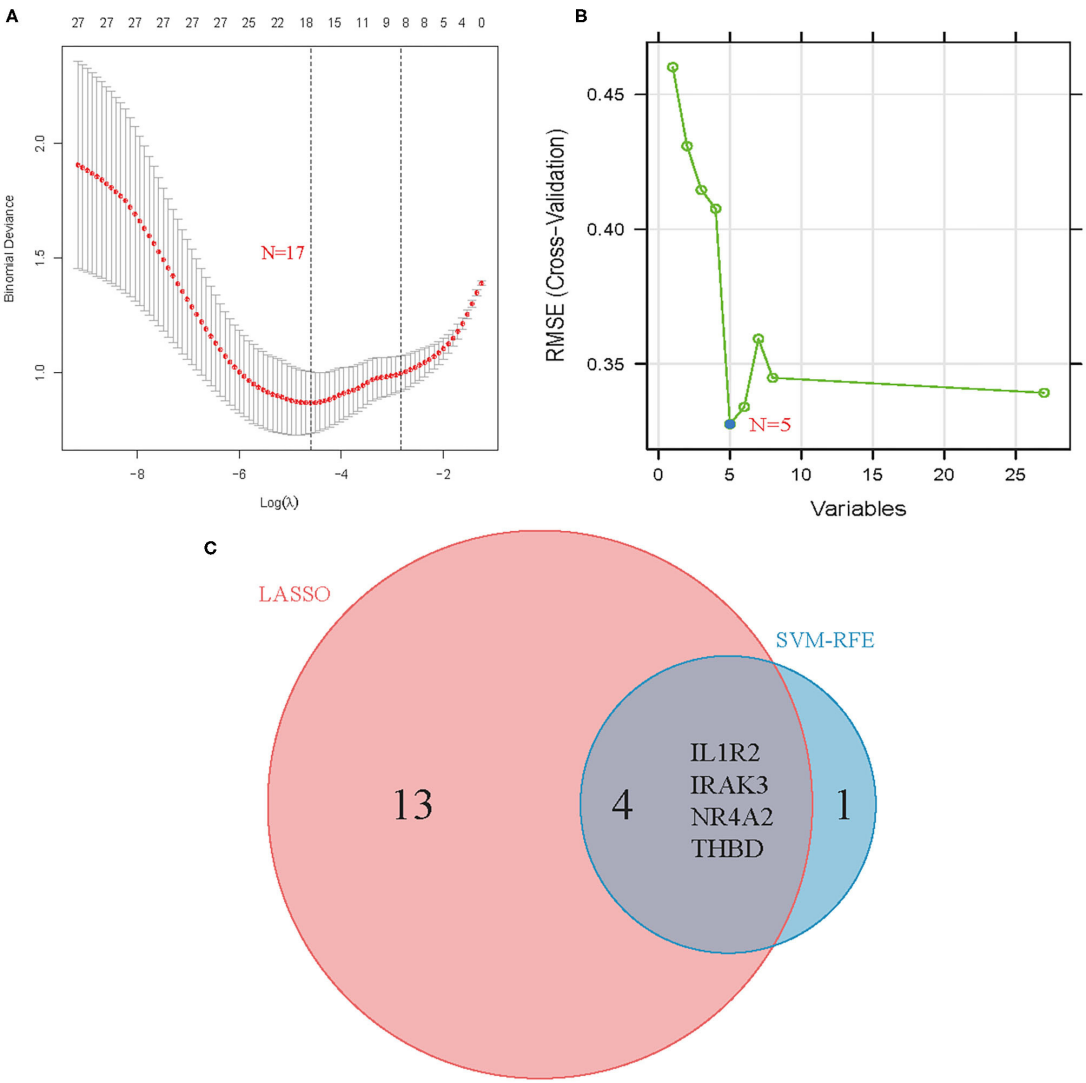
Screening process of diagnostic biomarker candidates for acute myocardial infarction diagnosis. **(A)** Tuning feature selection in the least absolute shrinkage and selection operator model. **(B)** A plot of biomarkers selection via support vector machine-recursive feature elimination (SVM-RFE) algorithm. **(C)** Venn diagram demonstrating four diagnostic markers shared by the least absolute shrinkage and selection operator and SVM-RFE algorithms.

**Figure 4:**
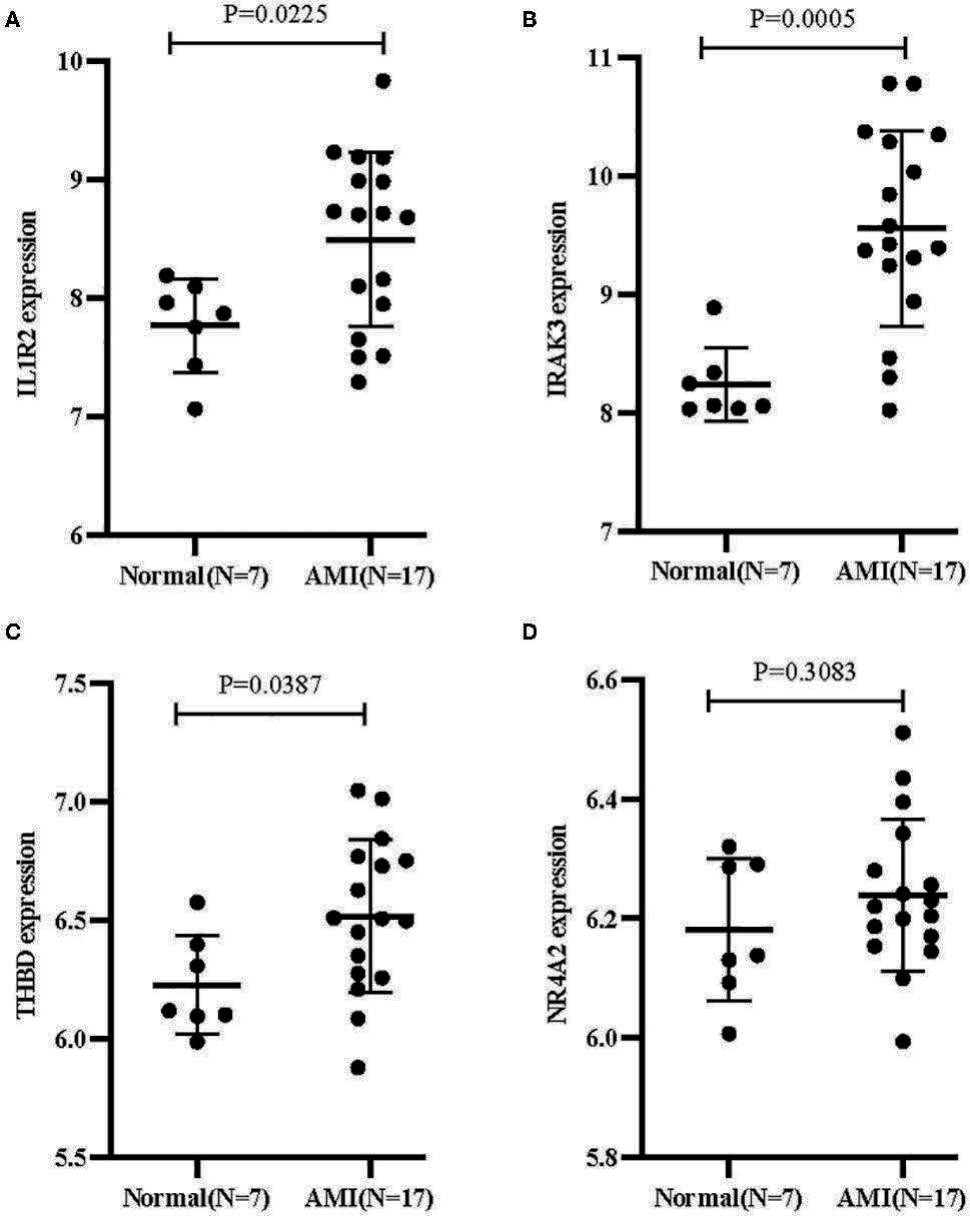
Validation of the expression of diagnostic biomarkers in the GSE60993 dataset. **(A)** IL1R2; **(B)** IRAK3; **(C)** THBD; **(D)** NR4A2.

Two different algorithms were used to screen potential biomarkers. The DEGs were narrowed down using the LASSO regression algorithm, resulting in the identification of 17 variables as diagnostic biomarkers for AMI (Figure 3A). A subset of five features among the DEGs was determined using the SVM-RFE algorithm (Figure 3B). The four overlapping features (IL1R2, IRAK3, NR4A2, and THBD) between these two algorithms were ultimately selected (Figure 3C). Furthermore, to generate more accurate and reliable results, the GSE60993 dataset was used to verify the expression levels of the four features. The expression levels of IL1R2, IRAK3, and THBD in AMI tissue were notably higher than those in the control group (Figures 4A–C; all *P* < 0.05). However, there was no significant difference between the two groups in terms of THBD expression (Figure 4D). Therefore, the three identified genes were used to establish the diagnostic model using a logistic regression algorithm in the metadata cohort.

### Diagnostic Effectiveness of Feature Biomarkers in AMI

**Figure 5:**
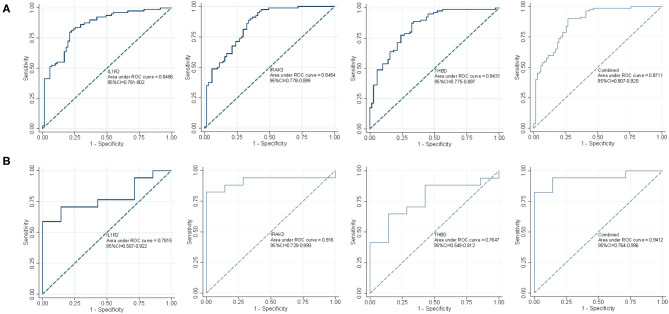
The receiver operating characteristic (ROC) curve of the diagnostic effectiveness of the three diagnostic markers. **(A)** ROC curve of IL1R2, IRAK3, and THBD after fitting to one variable in the metadata cohort; **(B)** ROC curve of IL1R2, IRAK3, and THBD after fitting to one variable in the GSE60993 dataset.

As shown in Figure 5A, the diagnostic ability of the three biomarkers in discriminating AMI from the control samples demonstrated a favorable diagnostic value, with an AUC of 0.849 (95% CI 0.781–0.902) in IL1R2, AUC of 0.845 (95% CI 0.778–0.899) in IRAK3, and AUC of 0.843 (95% CI 0.775–0.897) in THBD. When the three genes were combined into one variable, the diagnostic ability in terms of AUC was 0.871 (95% CI 0.807–0.920) in the meta-data cohort. Moreover, a powerful discrimination ability was confirmed in the GSE60993 dataset with an AUC of 0.782 (95% CI 0.567–0.922) in IL1R2, AUC of 0.916 (95% CI 0.729–0.990) in IRAK3, and AUC of 0.765 (95% CI 0.549–0.912) in THBD. Importantly, the diagnostic ability of the three biomarkers combined yielded an AUC of 0.941 (95% CI 0.764–0.996; Figure 5B), indicating that the feature biomarkers had a high diagnostic ability.

### Immune Cell Infiltration

**Figure 6:**
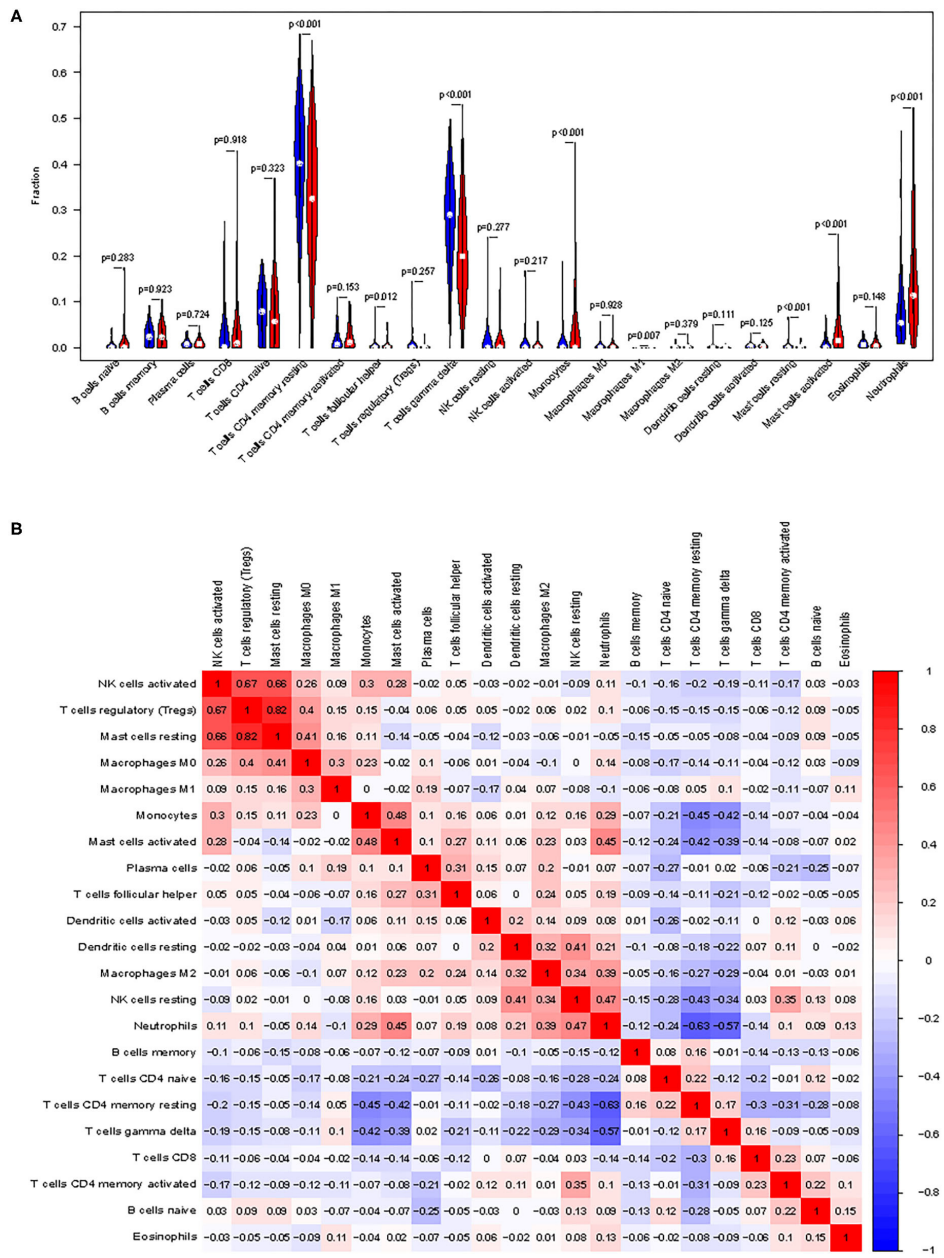
Distribution and visualization of immune cell infiltration. **(A)** Comparison of 22 immune cell subtypes between acute myocardial infarction tissues and normal tissues. Blue and red colors represent normal and acute myocardial infarction samples, respectively. **(B)** Correlation matrix of all 22 immune cell subtype compositions. Both horizontal and vertical axes demonstrate immune cell subtypes. Immune cell subtype compositions (higher, lower, and same correlation levels are displayed in red, blue, and white, respectively).

First, we explored the composition of immune cells in AMI tissues vs. normal control tissues. The proportions of CD4^+^ resting memory T cells (*P* < 0.001), gamma delta T cells (*P* < 0.001), M1 macrophages (*P* = 0.007), and resting mast cells (*P* < 0.001) in AMI tissues were significantly lower than in normal tissues. However, the proportion of monocytes (*P* < 0.001), activated mast cells (*P* < 0.001), neutrophils (*P* < 0.001), and follicular helper T cells (*P* = 0.012) in AMI tissues was significantly higher than that in normal tissues (Figure 6A).

The correlation of 22 types of immune cells was calculated (Figure 6B). CD4 memory resting T cells were significantly positively correlated with memory B cells (*r* = 0.23, *P* = 0.042), but significantly negatively correlated with monocytes (*r* = −0.42, *P* = 0.023), activated mast cells (*r* = −0.43, *P* = 0.014), and neutrophils (*r* = −0.55, *P* < 0.001). Follicular helper T cells were significantly positively correlated with plasma cells (*r* = 0.42, *P* = 0.00011) and regulatory T cells (*r* = 0.42, *P* = 0.0247), but significantly negatively correlated with CD8 T cells (*r* = −0.31, *P* = 0.0051). Gamma delta T cells were significantly positively correlated with CD8 T cells (*r* = 0.22, *P* = 0.046), but significantly negatively correlated with monocytes (*r* = −0.44, *P* = 0.0092), activated mast cells (*r* = −0.44, *P* = 0.011), and neutrophils (*r* = −0.55, *P* < 0.001). Monocytes were significantly positively correlated with follicular helper T cells (*r* = 0.29, *P* = 0.0084) and mast cell activated (*r* = 0.43, *P* = 0.016), but significantly negatively correlated with CD8 T cells (*r* = −0.29, *P* = 0.0083), CD4 memory resting T cells (*r* = −0.42, *P* = 0.0001), and gamma delta T cells (*r* = −0.44, *P* < 0.0001). M1 macrophages were significantly positively correlated with gamma delta T cells (*r* = 0.25, *P* = 0.027) and resting dendritic cells (*r* = 0.49, *P* = 0.0008). Resting mast cells were significantly positively correlated with M0 macrophages (*r* = 0.26, *P* = 0.018). Activated mast cells were significantly positively correlated with follicular helper T cells (*r* = 0.31, *P* = 0.0042), activated NK cells (*r* = 0.43, *P* < 0.0001), monocytes (*r* = 0.42, *P* < 0.0001), and M2 macrophages (*r* = 0.34, *P* = 0.0017), but significantly negatively correlated with CD8 T cells (*r* = −0.23, *P* = 0.037), CD4 memory resting T cells (*r* = −0.43, *P* < 0.0001), gamma delta T cells (*r* = −0.43, *P* < 0.0001), and M0 macrophages (*r* = −0.22, *P* = 0.043). Neutrophils were significantly positively correlated with activated NK cells (*r* = 0.22, *P* = 0.045), monocytes (*r* = 0.34, *P* = 0.0017), M2 macrophages (*r* = 0.26, *P* = 0.0181), and activated mast cells (*r* = 0.51, *P* < 0.0001), but significantly negatively correlated with CD8 T cells (*r* = −0.28, *P* = 0.011), CD4 memory resting T cells (*r* = −0.55, *P* < 0.0001), and T cells gamma delta (*r* = −0.54, *P* < 0.0001).

### Correlation Analysis Between the Three Biomarkers and Infiltrating Immune Cells

**Figure 7:**
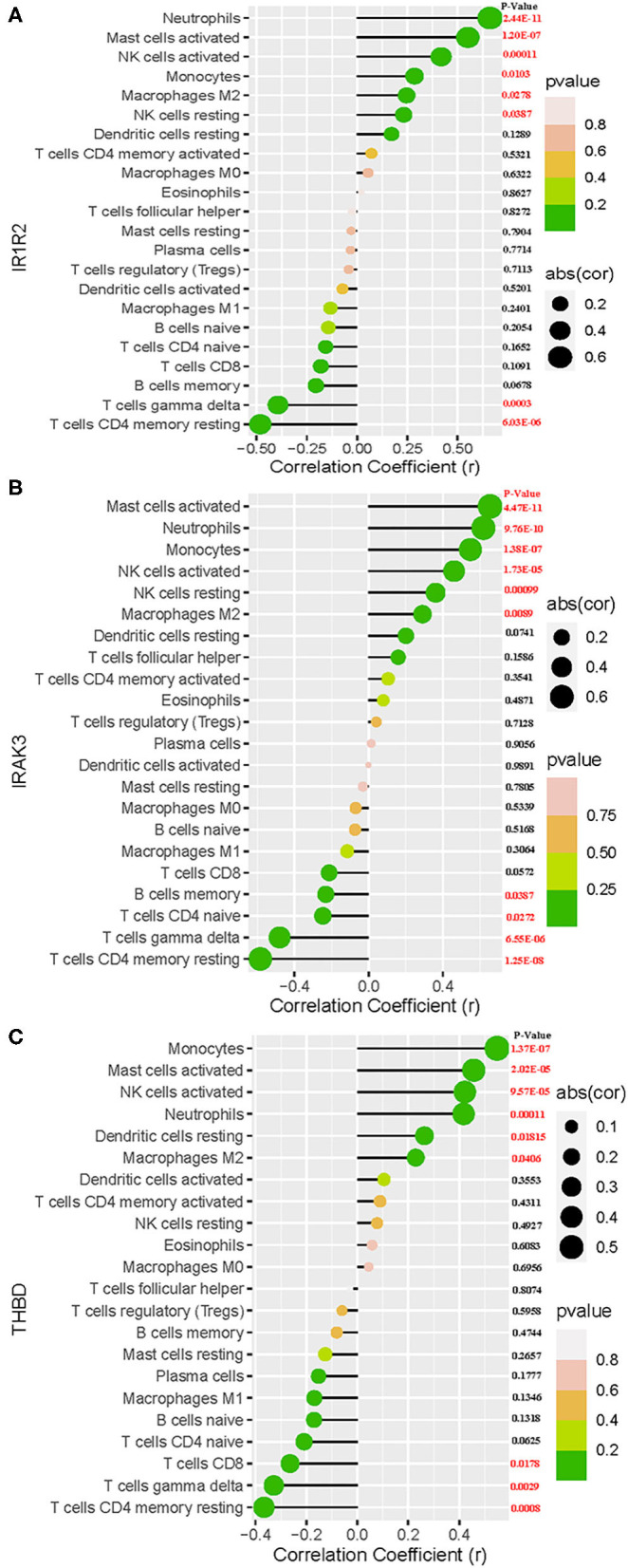
Correlation between IL1R2 **(A)**, IRAK3 **(B)**, THBD **(C)**, and infiltrating immune cells in acute myocardial infarction.

As shown in Figure 7A, IL1R2 was positively correlated with neutrophils (*r* = 0.66, *P* < 0.0001), activated mast cells (*r* = 0.55, *P* < 0.0001), activated NK cells (*r* = 0.42, *P* = 0.00011), monocytes (*r* = 0.28, *P* = 0.01), M2 macrophages (*r* = 0.25, *P* = 0.027), and resting NK cells (*r* = 0.23, *P* = 0.038) and negatively correlated with CD4 memory resting T cells (*r* = −0.48, *P* < 0.0001), and gamma delta T cells (*r* = −0.39, *P* = 0.00029). IRAK3 was positively correlated with activated mast cells (*r* = 0.65, *P* < 0.0001), neutrophils (*r* = 0.62, *P* < 0.0001), monocytes (*r* = 0.55, *P* < 0.0001), activated NK cells (*r* = 0.46, *P* < 0.0001), resting NK cells (*r* = 0.36, *P* = 0.001), and M2 macrophages (*r* = 0.29, *P* = 0.0089) and negatively correlated with CD4 memory resting T cells (*r* = −0.58, *P* < 0.0001), gamma delta T cells (*r* = −0.48, *P* < 0.0001), CD4 naïve T cells (*r* = −0.24, *P* = 0.027), and memory B cells (*r* = −0.23, *P* = 0.038; Figure 7B). THBD was positively correlated with monocytes (*r* = 0.54, *P* < 0.0001), activated mast cells (*r* = 0.45, *P* < 0.0001), activated NK cells (*r* = 0.42, *P* < 0.0001), neutrophils (*r* = 0.41, *P* = 0.00012), resting dendritic cells (*r* = 0.26, *P* = 0.018), and M2 macrophages (*r* = 0.23, *P* = 0.0418) and negatively correlated with CD4 memory resting T cells (*r* = −0.36, *P* = 0.0008), gamma delta T cells (*r* = −0.32, *P* = 0.0029), and CD8 T cells (*r* = −0.26, *P* = 0.0178; Figure 7C).

## Discussion

AMI remains a leading cause of mortality and disability despite great improvements in early diagnosis and treatment over the past decade (26). As a result, the clinical prognosis of patients with AMI is poor. Because of the lack of an effective early diagnosis, patients with AMI often lose the chance to benefit from treatment, resulting in poor outcomes. Recently, immune cell infiltration has been confirmed to play a vital role in the occurrence and development of AMI (20, 27, 28). Therefore, researchers are increasingly searching for novel diagnostic biomarkers and exploring the compositions of AMI immune cell infiltration, which could have a highly beneficial impact on the clinical outcomes of AMI patients. Recently, mRNAs and microRNAs have emerged as promising biomarkers in cardiovascular disease in general and in AMI in particular. For example, SOCS3 could serve as a biomarker to predict the risk of AMI, where the elevated expression of the SOCS3 gene is an independent risk factor for AMI (16). In particular, miR-34, which is known to modulate immunity, was found to be significantly modulated in post-MI heart failure, providing important information on its role in heart failure (29, 30). However, very few studies have focused on the aberrantly expressed gene biomarkers associated with immune infiltration between AMI and normal tissues. Therefore, we aimed to identify candidate diagnostic biomarkers for AMI and investigate the role of immune cell infiltration in AMI.

To the best of our knowledge, this is the first retrospective study to identify diagnostic biomarkers associated with immune cell infiltration in patients with AMI by mining multiple GEO datasets. We collected two cohorts from the GEO datasets and conducted an integrated analysis of the data. A total of 27 DEGs were identified, including 25 upregulated genes and 2 downregulated genes. The results of enrichment analyses indicated that diseases enriched by DEGs were mainly associated with atherosclerosis and arteriosclerotic cardiovascular disease. The GSEA results demonstrated that the enriched pathways generally involved inflammation and immune response pathways, such as cytokine–cytokine receptor interaction, atherosclerosis, and TNF signaling. These findings are in general agreement with the previous finding that an inflammatory response involving leukocytes participates in the pathogenesis of AMI (31). In fact, AMI is mainly caused by atherosclerosis and is regarded as a chronic inflammatory disorder (32). A substantial amount of inflammatory responses were induced during the acute phase of cardiac injury, caused by an abrupt cessation of blood flow, resulting in MI. The tumor necrosis factor (TNF) signaling pathway participates in inflammatory cell accumulation, platelet aggregation, vulnerable plaque formation, cardiomyocyte apoptosis, and poor remodeling after AMI (33). Cytokines, such as TNF and interleukin-8, have been confirmed to be involved in cell differentiation and inflammatory response via binding to specific receptors on the cell surface during the development of AMI (34). This evidence is consistent with our results, confirming that the findings in the present study are accurate, as well as demonstrating that the immune response plays a vital role in AMI. The significance of the immune system for cardiac repair after AMI is undeniable. Perhaps the most diverse and complex reaction after AMI is the immune response, which has been confirmed to influence various repair processes. Thus, a precise control over various types of immune cells is needed to achieve a safe and effective treatment (35). Therefore, the identification of novel biomarkers of AMI correlated with the magnitude of immune cell infiltration by bioinformatics analysis will contribute to its treatment.

Based on two machine-learning algorithms, three diagnostic markers were identified. Interleukin-1 (IL-1) is a major pro-inflammatory cytokine produced by smooth muscle cells, endothelial cells, and macrophages, which can stimulate the expression of genes related to inflammation and immunity. Interleukin-1 receptor type 2 (IL1R2), a cytokine receptor that belongs to the IL-1 receptor family, has been reported to serve as a critical mediator involved in many cytokines induced by immune and inflammatory responses (36). IL1R2 gene can control cell metabolism, as well as immune response induced by many cytokines (37). IL-1-mediated inflammation contributes to the pathology of many diseases including systolic heart failure, and IL-1R2 has been implicated in atherosclerosis (38). The aforementioned evidence suggests that IL1R2 plays a key role in AMI. Interleukin 1 receptor associated kinase 3 (IRAK3), which encodes a member of the IL-1 receptor-associated kinase protein family, functions as a negative regulator of Toll-like receptor signaling and participates in innate host defense and in the control of adaptive immune responses (39). Evidence in a mouse model of AMI demonstrated that IRAK3 gene silencing could minimize AMI damage, indicated by a reduced infarct area and collagen content (40). A mutation in the thrombomodulin (THBD) gene is the main cause of thromboembolic disease. AMI is typically precipitated by thrombosis superimposed on a ruptured coronary plaque. Therefore, we believe that THBD may play a vital role in the development of AMI.

The types of immune cell infiltration in AMI and normal samples were assessed using CIBERSOTR. As a result, a variety of immune cell subtypes were found to be closely involved in important biological processes of AMI. An increased infiltration of monocytes, activated mast cells, neutrophils, and T follicular helper cells, and a decreased infiltration of CD4^+^ resting memory T cells, gamma delta T cells, M1 macrophages, and resting mast cells were found to be potentially related to the occurrence and development of AMI. Furthermore, by performing correlation analysis between IL1R2, IRAK3, THBD, and immune cells, IL1R2, IRAK3, and THBD were all found to be correlated with neutrophils, monocytes, M2 macrophages, CD4^+^ resting memory T cells, gamma delta T cells, and activated NK cells. In fact, inflammatory and immune circulatory cells, such as neutrophils, lymphocytes, and platelets, have previously been shown to play an important role in the progression of heart disease (41, 42). The innate immune system begins immediately on the onset of necrotic cell death accompanied by intense sterile inflammation and the MI of a number of immune cell subtypes including monocytes and neutrophils during the first few days after AMI (28). Neutrophils can infiltrate the infarcted area, subsequently mediating the injury of infarcted tissues by releasing reactive oxygen species and matrix-degrading enzymes (43). CD4^+^ and CD8^+^ T cells, regulatory T cells, and NK T cells can infiltrate the infarcted myocardium during the proliferative phase of repair and facilitate the transition toward maturation. They may be motivated by cardiac autoantigens and limit adverse ventricular remodeling by enhancing wound healing, inflammation resolution, and scar development via collagen matrix formation (43). Furthermore, the therapeutic activation of regulatory T cells may well be an encouraging therapy for AMI to promote cardiac repair and limit adverse ventricular remodeling (44). The substantial evidence mentioned earlier together with our present findings have demonstrated that several types of infiltrating immune cells play vital roles in AMI and should be the focus of future investigations.

The limitations of this study should be acknowledged. First, the study was retrospective; thus, important clinical information was not available. Second, the number of cases in the GSE60993 validation cohort was low, which should be acknowledged as a limitation. In addition, the biomarker profiles in the blood and the immune cell profile were obtained from the two datasets, and their reproducibility should be further validated. Last, the functions of three biomarkers and immune cell infiltration in AMI were inferred by bioinformatics analysis, and prospective studies with larger sample sizes should be conducted to validate our conclusions.

## Conclusion

In summary, IL1R2, IRAK3, and THBD were identified as diagnostic biomarkers of AMI. Neutrophils, monocytes, M2 macrophages, CD4^+^ resting memory T cells, gamma delta T cells, and activated NK cells may be involved in the development of AMI. These immune cells have the potential to be developed as targets of immunotherapy in patients with AMI.

## Data Availability Statement

Publicly available datasets were analyzed in this study. This data can be found here: All the raw data used in this study are derived from the public GEO data portal (https://www.ncbi.nlm.nih.gov/geo/; Accession numbers: GSE66360, GSE48060, and GSE60993).

## Author Contributions

EZ and HX is the principal investigator and conducted statistical analysis and drafted the article. EZ performed data management and bioinformatics analysis. EZ, HX, and YZ edited and revised the article. All authors read and approved the final article.

## Conflict of Interest

The authors declare that the research was conducted in the absence of any commercial or financial relationships that could be construed as a potential conflict of interest.
